# Interactive effect of serum uric acid and total bilirubin for cardiovascular disease in Chinese patients with type 2 diabetes

**DOI:** 10.1038/srep36437

**Published:** 2016-11-02

**Authors:** Yanfeng Ren, Nan Jin, Tianpei Hong, Yiming Mu, Lixin Guo, Qiuhe Ji, Qiang Li, Xilin Yang, Linong Ji

**Affiliations:** 1 Department of Epidemiology and Biostatistics, School of Public Health, Tianjin Medical University, Tianjin, China; 2 Department of Health Statistics, School of Public Health and Management, Weifang Medical University, Shandong, China; 3 Department of Endocrinology, Chinese PLA General Hospital, Beijing, China; 4 Department of Endocrinology, Peking University Third Hospital, Beijing, China; 5 Department of Endocrinology, Beijing Hospital, Beijing, China; 6 Department of Endocrinology, The Fourth Military Medical University Xi Jing Hospital, Xi’an, China; 7 Department of Endocrinology, The 2^nd^ Affiliated Hospital of Harbin Medical University, Harbin, Heilongjiang, China; 8 Department of Endocrinology, Peking University People’s Hospital, Beijing, China

## Abstract

Serum uric acid (SUA) at high levels and bilirubin at low levels were potent antioxidant but it was uncertain that whether SUA and total bilirubin (TBIL) had additive interaction for the risk of CVD in type 2 diabetes mellitus (T2DM). We conducted a cross-sectional survey of 6713 inpatients with T2DM and admitted to 81 tertiary care hospitals. CVD was defined as having either prior coronary heart disease or stroke or peripheral arterial disease. Binary logistic regression was used to estimate odds ratios of SUA and TBIL for CVD. The effect size of additive interaction was estimated by three measures, i.e., relative excess risk due to interaction, attributable proportion due to interaction and synergy index. Among 6713 patients with T2DM, 561 (8.36%) suffered from CVD. Using ≥283 umol/L (median) to define high SUA and <11.5 umol/L (n = 2290 or 34.11%) to define low TBIL, copresence of both factors (n = 621 or 9.25%) was associated with 5.18-fold (95% CI, 4.00–6.72) risk of CVD with significant additive interactions in multivariable analysis as compared to absence of both risk factors. The copresence of both high SUA and low TBIL was associated with a large increased risk of CVD in high-risk Chinese patients with type 2 diabetes.

Cardiovascular disease (CVD) is one of the most severe complications of type 2 diabetes mellitus (T2DM) and a major cause of death among patients with T2DM^1^. With the escalating prevalence of T2DM worldwide^2^, CVD mortality in T2DM patients exceeds 60%^3^. Although major trials demonstrated that tight glycemia control^4^ and especially, multifactorial intervention^5^ were able to reduce the risk of CVD in T2DM, the residual risk of CVD remained high. In this connection, several groups developed risk scores for prediction of cardiovascular diseases in T2DM^6^,^7^ but the accuracy of these developed risk score is still suboptimal, e.g., the area under receiver’s operating characteristics curves being only 0.74 for Chinese patients with T2DM^7^. It is essential to further investigate novel risk factors for CVD and to look for potential ways to reduce the burden of CVD in T2DM.

In this regard, high uric acid levels and low bilirubin concentrations were observed to be associated with CVD^8^,^9^. Uric acid is a metabolic end product of purine metabolism in humans and possesses pro-oxidant and antioxidant dual properties. Higher levels of serum uric acid (SUA) may overwhelm its antioxidant function and play a pro-oxidant role in the body^10^. Previous epidemiological or experimental studies reported that elevated levels of SUA were associated with glucose metabolic disorders, i.e. impaired fasting glucose, elevated glycated hemoglobin (HbA1_c_), hyperinsulinemia and insulin resistance^11^,^12^ and diabetes^13^. In addition to glucose metabolic disorders, some studies demonstrated that higher levels of SUA were associated with dyslipidemia, hypertension, cardiovascular diseases and ischemic stroke^14^,^15^. Although the association between the elevated levels of SUA and the severity of metabolic syndrome and cardiovascular diseases were in a linear manner^14^,^16^, J-shaped and U-shaped associations with cardiovascular mortality were reported in two cohort studies^17^,^18^.

Bilirubin is an end product of heme metabolism by heme oxygenase, a key antioxidant enzyme, and excreted by liver cells. Traditionally, bilirubin was regarded as a toxic waste product. However, recent studies demonstrated that bilirubin has both anti-oxidative and anti-inflammatory properties in adults, and suppresses oxidization of lipids and lipoprotein^19^,^20^. Anti-oxidative activity of bilirubin is independent of its forms, i.e., unconjugated and conjugated bilirubins^21^. Several studies reported that serum bilirubin concentrations were inversely associated with the risk of homeostasis model assessment insulin resistance, diabetes, and metabolic syndrome^22^,^23^. Several prospective cohort studies found that high serum bilirubin may have cardiovascular benefits, such as atherosclerosis, stenosis, stroke, and myocardial infarction^24^,^25^. In a recent statin-treated cohort of 130,052 patients, total serum bilirubin (TBIL) was associated with the risk of CVD in a L-shaped manner^9^. However, another prospective epidemiological study reported a U-shaped relationship between bilirubin levels and the risk of coronary heart disease^26^.

Given to the anti-oxidative and anti-inflammatory properties of SUA and serum bilirubin and their associations with morbidities (e.g., diabetes and CVD), it is worthwhile to investigate whether low bilirubin and high SUA levels can enhance the risk of each other for CVD when co-existing, especially in high-risk groups with T2DM. The current study used the data from a cross sectional survey of inpatients with T2DM from 81 top tertiary care hospitals in China to test 1) full-range associations of SUA and TBIL with CVD; and 2) interactive effects of both factors towards increasing the risk of CVD in Chinese patients with T2DM.

## Methods

### Patients

From May 2013 to August 2013, a survey as a quality improvement program was conducted by Chinese Hospital Association in patients with T2DM from top tertiary hospitals in China to learn the profile of management of Chinese with T2DM, A total of 81 top tertiary care hospitals in 27 cities from 21 Chinese provinces were invited and agreed to participate in the survey. The inclusion criteria were 1) With T2DM and admitted to the department of endocrinology; 2) Agreed to use management scheme of basal bolus plus meal time insulin intensive treatment after admission; and 3) Aged between 18 to 80 years. The exclusion criteria were 1) With liver dysfunction defined as alanine aminotransferase or aspartate aminotransferase ≥2.5 folds of the upper limits of the normal range, 0–40 U/L; 2) With renal dysfunction defined as serum creatinine ≥125 μmol/L in male and ≥110 μmol/L in female or chronic kidney disease; 3) During pregnancy or lactation; and 4) Unable to communicate in a normal way.

During the survey period, a total of 6713 inpatients with T2DM were consecutively recruited from the department of endocrinology of 81 participating top tertiary care hospitals. All the 6713 patients were used in the current analysis because there were no missing values in the key variables, i.e., SUA and TBIL. The survey was conducted in accordance with the guidelines of the Declaration of Helsinki and approved by the ethics committee of the People’s General Army Hospital Clinical Research Ethics Committee. Written informed consent was obtained before the data collection.

### Data collection and clinical measurements

Postgraduate medical students or research nurses were trained to do fieldworkers. The fieldworkers reviewed registers of the inpatients and retrieved the demographic information and the results of clinical measurements and laboratory measurements. The collected parameters included age, duration of diabetes, gender, body weight, body height, and sitting blood presure (BP), self-monitoring of blood glucose (SMBG), glycated haemoglobin(HbA_1c_), lipid profile, SUA, TBIL, liver function and renal fucntion. Fasting blood was taken for measurement of HbA_1c_, SUA, TBIL and lipids including low-density lipoprotein cholesterol (LDL-C), high-density lipoprotein cholesterol (HDL-C), triglyceride (TG). The first morning urine was used to essay albuminuria and creatinine, and then urinary albumin to creatinine ratio (ACR) was calculated. Use of drugs was documented in details, including lipid lowering drugs (statins and other lipid lowing drugs), antihypertensive drugs (renin angiotensin system inhibitors, calcium antagonists and β- receptor antagonists) and antidiabetes drugs (oral antidiabetes drugs, glucagon-like peptide-1 based drugs and insulin and combinations of these drugs). Diabetes micro- and macro- vascular complications were also documented in details. Macro-vascular complications included ischemic heart disease, myocardial infarction, coronary revascularization, coronary atherectom, or percutaneous transluminal coronary angioplasty, stroke, lower limb amputation, revascularization for peripheral arterial disease and absence of foot pulses. Sensory neuropathy, diabetic nephropathy and diabetic retinopathy were also documented.

### Definition of cardiovascular disease

Medical records from secondary or tertiary hospitals were used to define cardiovascular disease. Previous coronary heart disease included myocardial infarction with typical changes in electrocardiogram and plasma enzyme testing, ischaemic heart disease with abnormal electrocardiogram or stress test, percutaneous transluminal coronary angioplasty, coronary revascularisation, or coronary atherectomy. Previous stroke prior stroke (but not transient ischemic attack) included ischaemic or haemorrhagic stroke, i.e., intracerebral haemorrhage, subarachnoid haemorrhage, and other or unspecified intracranial haemorrhage, no matter whether the stroke was completely or incompletely recovered. Peripheral arterial disease (PAD) was defined by lower limb amputation, revascularization for PAD or absence of foot pulses (ankle-brachial ratio <0.90 measured by Doppler ultrasound evaluation). In this study, CVD was defined as having either CHD or stroke or PAD.

### Statistical Analysis

The Statistical Analysis System (SAS, version 9.3; SAS Institute, Inc., Cary, NC, USA) was used to analyze the data. Continuous variables were expressed as means and standard deviations or medians and interquartiles. Categorical variables were expressed by numbers and percentages. Normality test was used to check the distribution of continuous variables. Categorical variables between patients with CVD and without CVD were compared using Chi-square test or Fisher’s exact test where appropriate. Continuous variables between two groups were compared by student t test if normal distribution was not rejected or two-sample Wilcoxon rank test otherwise. Binary logistic regression was used to estimate odds ratios of TBIL and SUA for CVD. A structured adjustment scheme was used to adjust for confounding effects of other variables. First, we estimated ORs for CVD in univariable logistic analysis. Then, we performed multivariable analysis and obtained adjusted ORs of TBIL and SUA for CVD, with adjustment for demographic and clinical factors, complications of diabetes, and use of drugs. Adjusted variable included age, duration of diabetes, gender, body mass index, systolic blood pressure, diastolic blood pressure, HbA_1c_, LDL-C, HDL-C, triglyceride, SMBG, log-transformed ACR(6 patients with data missing for ACR), complications of diabetes, blood pressure- and lipid- lowing and anti-diabetic drugs. We also performed sensitivity analysis in patients with diabetes duration ≥2 years to check the impacts of occurrence of CVD in early stage of T2DM.

Some previous studies reported inconsistent curvilinear relations manner between SUA, TBIL and CVD^9^,^17^,^18^,^26^. Initially, we stratified SUA and TBIL into quartiles in examining their associations with CVD (Supplementary Table 1). To capture full range relationship patterns of SUA and TBIL with CVD, we further used restricted cubic splines (RCS) in binary logistic regression^27^ and plotted ORs against a chosen reference level of these two factors to show their full-range associations with CVD as used before^28^. Four knots (5%, 35%, 65% and 95%) were used in the analysis as suggested by Harrell^27^. We stratified the two continuous variables into binary ones at cutoff points obtained by visual checking of the shapes of those curves, i.e., where the OR started to rise or to decrease rapidly^28^ for subsequent data analysis of possible additive interactions.

Initially, the histogram was used to check the possible additive interaction. As we found that there was a high percent of CVD cases in the group with co-exposures to high SUA and low TBIL as compared to exposure to either or non-exposure to any of them (Supplementary Figure 1). We calculated relative excess risk due to interaction (RERI), attributable proportion due to interaction (AP) and synergy index (S)^29^ to estimate effect sizes of additive interactions. As before^30^, we defined significant additive interaction as any of the three measures being significant in this analysis. RERI > 0, AP > 0, or S > 1 indicates additive interaction between two variables. Peril ratio index of synergy based on multiplicativity (PRISM) was also performed to check consistency by different methods examining interactions between two risk factors. PRISM > 1 indicates a positive interaction between two variables^31^. P values less than 0.05 from two tailed tests were considered to be statistically significant.

## Results

### Characteristics of the study patients

Mean age of inpatients was 56.38 (standard deviation: 10.55) years with a median of 3.00 (interquartile: 0.41 to 6.05) years of duration of diabetes. Female patients accounted for 43.44%. Among them, 561 (8.36%) suffered from CVD (CHD: 357 or 5.32%; stroke: 106 or 1.58%; PAD: 216 or 3.21%). Patients with CVD were more likely to have an older age, longer duration of diabetes, higher BMI, HbA1_c_, and BPs, and more frequent use of SMBG than those without CVD. LDL-C and TG were higher but HDL-C and ACR were lower in patients with CVD than in those without CVD. Patients with CVD were more likely to have almost all diabetes complications of micro-vascular disease, and more likely to use statins, renin angiotensin system inhibitors and antidiabetes drugs including oral antidiabetes drugs (OADs), glucagon-like peptide-1(GLP-1) based drugs and insulin than those without CVD (Table 1).

### Associations of SUA and TBIL with CVD

**Figure 1:**
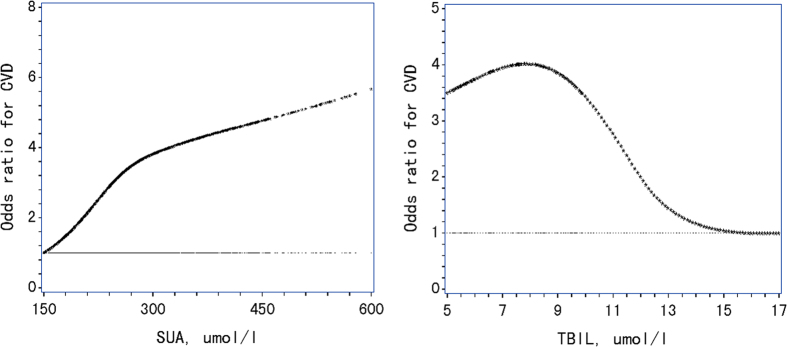
Odds ratio curves SUA and TBIL for CVD in Chinese patients with type 2 diabetes. The curves were derived from multivariate models adjusted for age, duration of diabetes, gender, body mass index, systolic blood pressure, diastolic blood pressure, glycated hemoglobin, low-density lipoprotein cholesterol, high-density lipoprotein cholesterol, triglyceride, self-monitoring, log-transformed urinary albumin to creatinine ratio, and drug use and complications as listed in Table 1 and spline functions of SUA, and TBIL were used to adjust for their non-linear confounding effects; SUA, serum uric acid; TBIL, total bilirubin; CVD, cardiovascular disease.

SUA was associated with the risk of CVD in a roughly linear manner. The risk of CVD rapidly increased with increasing SUA level up to 283 umol/l (i.e., its median), and then slightly leveled off. TBIL was associated with CVD in a roughly S-shaped manner with a zenith at 8 umol/l and a nadir at 14 umol/l. The CVD risk slightly increased from 14 umol/l umol/l to 11.5 umol/l and then sharply increased since 11.5 umol/l and downwards until 8 umol/l (Fig. 1).

If SUA and TBIL were categorized into two binary variables at their cutoff points of ORs increasing rapidly, i.e., 283 umol/l (SUA) and 11.5 umol/l (TBIL), high SUA was associated with increased risk for CVD in univariable model (OR: 1.47, 95%CI: 1.23–1.75) and in multivariable model (OR: 1.49, 95%CI: 1.25–1.78). Similarly, low TBIL was also associated with increased risk for CVD in univariable model (OR: 2.22, 95%CI: 1.86–2.64) and in multivariable model (OR: 2.15, 95%CI: 1.80–2.56) (Table 2).

### Subgroup effects and interactive effects of SUA and TBIL on CVD

The ORs of high versus low SUA were highly significant among patients with TBIL <11.5 umol/l (univariable OR: 5.21, 95%CI: 4.02–6.81; multivariable OR: 5.25, 95%CI: 4.06–6.81) but the ORs of high versus low SUA was greatly attenuated and became non-significant among patients with TBIL ≥ 11.5 umol/l (univariable OR: 0.82, 95%CI: 0.64–1.05; multivariable OR: 0.80, 95%CI: 0.62–1.04). Similarly, the ORs of low versus high TBIL were highly significant among patients with SUA ≥ 283 umol/l (univariable OR: 6.44, 95%CI: 5.08–8.18; multivariable OR: 6.45, 95%CI: 5.06–8.23) but non-significant among patients with SUA <283 umol/l in univariable analysis (OR: 1.01, 95%CI: 0.78–1.33) and in multivariable analysis (OR: 0.97, 95%CI: 0.74–1.27) (Table 2).

In the formal testing of additive interaction between SUA and TBIL for CVD, presence of SUA ≥283 umol/l alone was not associated with increased risk of CVD in univariable and multivariable analysis. In the same way, presence of TBIL ≤11.5 umol/l alone was also not associated with increased risk of CVD in univariable and multivariable analysis. On the other hand, copresence of both factors was associated with 5.30-fold risk (95% CI, 4.10–6.86) of CVD in univariable analysis and 5.18-fold (95% CI, 4.00–6.72) risk of CVD in multivariable analysis as compared to absence of both risk factors (Table 3). The measures for testing additive interactions were significant in both univariable analysis (RERI: 4.46, 95%CI: 3.30–5.62; AP: 0.84, 95%CI: 0.77–0.90) and multivariable analysis (RERI: 4.40, 95%CI: 3.25–5.54; AP: 0.84, 95%CI: 0.78–0.91) (Table 4). The measure of PRISM also showed a positive interaction between high SUA and low TBIL(PRISM: 1.30, 95%CI: 1.25–1.36) (Supplementary Table 2).

In addition, sensitivity analysis showed that the effect sizes of SUA and TBIL for CVD were similar in patients with diabetes duration ≥2 years (Supplementary Table 3). The AP, RERI and S were all significant (Supplementary Table 4). The detected associations between SUA, TBIL and their interactive effect with CVD were mainly driven by CHD, and to a lesser extent, by PAD (Supplementary Table 5 and Supplementary Table 6).

## Discussion

In this cross-sectional study of inpatients with type 2 diabetes, we used non-linear approach to define cutoff points for SUA and TBIL and found that presence of isolated high SUA, i.e., ≥283 umol/l and isolated low TBIL, i.e., <11.5 umol/l were not associated with increased risk of CVD. On the other hand, these two factors had an additive interaction towards increasing the risk of CVD and copresence of both factors was associated with a large risk of CVD in patients with type 2 diabetes.

Several prospective studies consistently demonstrated the positive relationship between SUA level and risk of cardiovascular diseases in general populations^8^,^15^. However, the association was inconsistent in patients with diabetes^32^,^33^,^34^. Although two studies reported that SUA was not a predictor for CVD mortality or all-cause mortality^34^,^35^, some studies found that high SUA was associated with increased risk of incident CVD or its components^32^,^33^. For example, a cross-sectional study showed that elevated uric acid level was a significant and independent risk factor for PAD in patients with T2DM (OR: 2.73, 95% CI: 1.23–6.04)^32^. A population-based 7-year follow-up study demonstrated that hyperuricemia was an independent predictor for fatal and nonfatal stroke in middle-aged patients with T2DM (HR: 1.93, 95% CI: 1.30–2.86)^33^. In a cohort study of 2726 outpatients with T2DM followed for a median of 4.7 years, elevated serum uric acid levels were independently increased the risk of cardiovascular mortality (HR: 1.27, 95% CI: 1.01–1.61)^36^. A prospective population-based study in 581 patients aged ≥65 years with T2DM showed a J-shaped association between SUA and mortality due to CHD^18^ although some authors argued that SUA was only a marker rather than a cause of CVD mortality in patient with T2DM^34^,^35^. Consistent with these studies^33^,^36^, our study found that elevated SUA was associated with increased risk of CVD in patients with T2DM, roughly in a linear manner.

Many cross sectional and cohort studies demonstrated that TBIL was negatively associated with CVD in general populations^9^,^24^,^25^,^26^. However, the association between TBIL and cardiovascular diseases has not been well investigated among patients with T2DM. A cross sectional study of 1,711 patients with T2DM showed that low TBIL levels were significantly associated with arterial stiffness (measured by brachial-ankle pulse wave velocity) in women but not in men^37^. The Fenofibrate Intervention and Event Lowering in Diabetes study identified a significant inverse association between bilirubin levels and amputation events, a direct consequence of PAD in T2DM^38^. Consistent with these findings, our study provided data regarding the negative association between TBIL levels and risk of CVD. In general people, TBIL was reported to be associated with CVD in a L-shaped and a U-shaped manner^9^,^26^. With use of the RCS curves, we found that TBIL was associated with the risk of CVD in a S-shaped manner in patients with T2DM.

To our knowledge, our study was the first reporting a synergistic effect between SUA and TBIL for the risk of CVD in patients with T2DM. In our study, patients co-existing high SUA and low TBIL had increased risk of CVD five times greater than those with low SUA and high TBIL. On the other hand, the risks of traditional factors (e.g., hypertension and hyperglycemia, etc.) on CVD were generally less than two times in previous studies^6^,^7^. Our finding offered evidence that patients with both high SUA level and low TBIL level were a more susceptible group at high risk of development of CVD than those with either of two risk factors or without any of the two risk factors. Our findings also offer an explanation why many other studies failed to detect significant associations of SUA and TBIL with CVD, i.e, not taking the interaction of these two factors for CVD into consideration.

Several *in vitro* and *in vivo* studies showed that reduced nitric oxide production and increased production of reactive oxygen species induced and promoted oxidative stress and endothelial dysfunction^39^,^40^, which are considered to play an important role in the pathogenesis of CVD, from the initiation of fatty streak to ultimate plaque rupture^39^,^41^. Hyperglycemia has a potent effect on increasing oxidation of low density lipoprotein^42^ and can activates renin-angiotensin system^43^, and the latter induces oxidative stress and inflammatory cascade^43^. In this regard, uric acid had the capacity of exerting pro-oxidant effects and decreasing nitric oxide bioavailability, and the association between hyperuricemia and cardiovascular disease can be explained by the property^44^. On the other hand, bilirubin as a potent endogenous antioxidant can bind free oxygen radicals under transformation to biliverdin and reduce levels of oxidative stress^20^, and thus, low TBIL level may contribute to the occurrence and development of CVD. Therefore, we speculate that the synergistic effect of the two factors for CVD in T2DM may stem from imbalance of oxidative and anti-oxidative system, which results in vascular damage, especially at presence of oxidative stress and activated inflammatory cascade induced by hyperglycemia. It is warranted to further investigate whether the increased risk of CVD with co-presence of high SUA and low TBIL is medicated via increased oxidative stress and inflammation in T2DM.

In addition to mechanistic implications, our findings also have important clinical importance. SUA and TBIL are routinely measured in many parts of the world as their usefulness in clinical practice, and may be useful markers for prediction of CVD when co-presence of high SUA and low TBIL. This group of patients may be at particular high risk of CVD, so copresence of both factors may be useful for stratification of CVD risk among patients with type 2 diabetes, individually or used in the predicting models such as Framingham heart score or Hong Kong Chinese CHD risk score^6^,^7^. Given to the importance of CVD in patients with T2DM^1^ and that this group accounted for 9.25% of the patients in the survey, it is needed to further investigate whether multifaceted intervention achieving the BP, lipid and glycemia control targets can reduce the increased risk of CVD associated with copresence of these two factors.

Certain limitations should be considered. First, this study was a cross-sectional study. Our findings need further replication studies in other populations, preferably, using cohort study designs and if confirmed, randomized controlled trials. Mechanistic investigations are also needed to understand the molecular mechanisms responsible for our observations. Second, the present study was a hospital-based study of inpatients with T2DM. The result may not be extrapolated to general population, even in patients with lighter T2DM. Third, CVD events in our study were indentified by reviewing medical records and some CVD cases may have been missing. Missing of CVD cases may lead to underestimate the association between variables of interest and CVD. Fourth, CVD events before T2DM were unknown and not excluded. However, our findings are robust, not confounded by CVD events occurred in early stage of T2DM as evidenced by the persistent significance of the additive interaction measures after excluding those patients within 2 years of diagnosed T2DM. Fifth, lifestyle and social-economic factors, such as smoking, alcohol drinking habits, education level, income, etc., were not collected in this survey. We do not ascertain whether these factors have effects on the association established in present study. Sixth, inpatients with abnormal liver and kidney function were excluded in present study. It was uncertain whether these associations were also robust among patients with these conditions.

In conclusion, copresence of SUA ≥ 283 umol/l and TBIL < 11.5 umol/l was associated with large increased risk of CVD in Chinese patients being hospitalized for type 2 diabetes. The novel risk factors may be useful to identify patients who are at particular high risk of CVD and therefore may be useful for prediction of CVD in type 2 diabetes. It is warranted to further investigate whether the increased risk associated with copresence of both factors is mediated via oxidative stress and inflammation. These findings are obtained from a single study of high-risk Chinese patients with type 2 diabetes, and thus, they need be confirmed in other populations, especially in cohorts of low risk patients with type 2 diabetes. It remains to see whether intensive intervention can reduce the increased risk of CVD associated with copresence of both factors.

## Additional Information

**How to cite this article**: Ren, Y. *et al*. Interactive effect of serum uric acid and total bilirubin for cardiovascular disease in Chinese patients with type 2 diabetes. *Sci. Rep.* **6**, 36437; doi: 10.1038/srep36437 (2016).

**Publisher’s note**: Springer Nature remains neutral with regard to jurisdictional claims in published maps and institutional affiliations.

## Supplementary Material

Supplementary Information

## Figures and Tables

**Table 1:** Demographic, Clinical and biochemical characteristics of subjects by CVD.

variable	Non CVD (n = 6152)	CVD (n = 561)	P value
	Median (25^th^ to 75^th^) or n (%)	Median (25^th^ to 75^th^) or n (%)	
Age, years	56 (50–64)	60 (53–65)	<0.0001*
Female gender	2662 (43.27)	254 (45.28)	0.3589*
Duration of diabetes, years	2.89 (0.35–5.90)	6.18 (3.98–9.99)	<0.0001*
Body mass index, kg/m^2^	23.78 (21.77–25.65)	24.22 (22.48–26.19)	0.0002*
HbA1_c_, %	10.20 (9.60–11.10)	10.40 (9.60–11.46)	0.0010†
HbA1_c_, mmol/l	88 (81–98)	90 (81–102)	0.0010†
Systolic blood pressure, mmHg	130 (123–135)	130 (128–140)	<0.0001*
Diastolic blood pressure, mmHg	80 (75–85)	82 (80–90)	<0.0001*
LDL-C, mmol/L	3.00 (2.69–3.70)	3.30 (2.60–3.56)	0.8507†
HDL-C, mmol/L	1.90 (1.43–2.90)	1.31 (1.09–2.40)	<0.0001†
TG, mmol/L	2.30 (1.54–3.00)	2.35 (1.80–2.76)	0.3042†
ACR, mg/mmol‡	0.17 (0.15–0.19)	0.16 (0.15–0.18)	0.0002†
SMBG, yes	1880 (30.56)	235 (41.89)	<0.0001*
Serum uric acid, umol/l	276.0 (223.0–340.0)	284.5 (258.0–344.5)	<0.0001†
≥283 umol/l	3031 (49.27))	330 (58.82)	<0.0001*
Total bilirubin, umol/l	12.10 (10.80–12.90)	11.40 (8.20–12.80)	<0.0001†
<11.5 umol/l	2000 (32.51)	290 (51.69)	<0.0001*
**Complications**			
Sensory neuropathy	214 (3.48)	216 (38.50)	<0.0001*
Diabetic nephropathy	130 (2.11)	90 (16.04)	<0.0001*
Diabetic retinopathy	132 (2.15)	149 (26.56)	<0.0001*
**Drug use**			
Stains	225 (3.66)	119 (21.21)	<0.0001*
Other lipid lowering drugs	46 (0.75)	18 (3.21)	<0.0001*
Renin-angiotensin system inhibitors	216 (3.51)	182 (32.44)	<0.0001*
Other antihypertensive drugs	105 (1.71)	100 (17.83)	<0.0001*
OADs only	2372 (38.56)	269 (47.95)	<0.0001*
GLP-1 based drugs alone or combined with OADs	19 (0.31)	8 (1.43)	<0.0001*
Insulin alone or combined with OADs	2053 (33.37)	233 (41.53)	<0.0001*

CVD, cardiovascular disease; HbA1_c_, glycated haemoglobin; SMBG, self monitoring of blood glucose; LDL-C, low-density lipoprotein cholesterol; HDL-C, high-density lipoprotein cholesterol; TG, triglyceride; ACR, urinary albumin to creatinine ratio; OADs, oral antidiabetes drugs; GLP, glucagon-like peptide.

^*^P values were derived from Chi-square test or t-test.

^†^P values were derived from wilcoxon rank test.

^‡^Validation sample size = 6152 for non-CVD and 555 for CVD.

**Table 2:** Odds ratios of SUA and TBIL for CVD in type 2 diabetes.

Exposures	N (%) of CVD‡	OR (95% CI)	P value
All patients			
Model 1*: SUA ≥283 umol/l vs. <283 umol/l	330 (9.82):231 (6.89)	1.47 (1.23,1.75)	<0.0001
Model 2†: SUA ≥283 umol/l vs. <283 umol/l	330 (9.82):231 (6.89)	1.49 (1.25,1.78)	<0.0001
Model 1*: TBIL < 11.5 umol/l vs. ≥11.5 umol/l	271 (6.13):290 (12.66)	2.22 (1.86,2.64)	<0.0001
Model 2†: TBIL < 11.5 umol/l vs. ≥11.5 umol/l	271 (6.13):290 (12.66)	2.15 (1.80,2.56)	<0.0001
Model 3*: TBIL ≥ 11.5 umol/l vs. <11.5 umol/l	290 (12.66):271 (6.13)	0.45 (0.37,0.53)	<0.0001
Model 4†: TBIL ≥ 11.5 umol/l vs. <11.5 umol/l	290 (12.66):271 (6.13)	0.46 (0.39,0.55)	<0.0001
Among patients with TBIL ≥ 11.5 umol/l			
Model 1*: SUA ≥283 umol/l vs. <283 umol/l	156 (5.69):115 (6.83)	0.82 (0.64,1.05)	0.1254
Model 2†: SUA ≥283 umol/l vs. <283 umol/l	156 (5.69):115 (6.83	0.80 (0.62,1.04)	0.0984
Among patients with TBIL < 11.5 umol/l			
Model 1*: SUA ≥283 umol/l vs. <283 umol/l	174 (28.02):116 (6.95)	5.21 (4.02,6.81)	<0.0001
Model 2†: SUA ≥283 umol/l vs. <283 umol/l	174 (28.02):116 (6.95)	5.25 (4.06,6.81)	<0.0001
Among patients with SUA < 283 umol/l			
Model 1*: TBIL < 11.5 umol/l vs. ≥11.5 umol/l	116 (6.95):115 (6.83)	1.01 (0.78,1.33)	0.8934
Model 2†: TBIL < 11.5 umol/l vs. ≥11.5 umol/l	116 (6.95):115 (6.83)	0.97 (0.74,1.27)	0.8602
Model 3*: TBIL ≥ 11.5 umol/l vs.<11.5 umol/l	115 (6.83):116 (6.95)	0.98 (0.75,1.28)	0.8934
Model 4†: TBIL ≥ 11.5 umol/l vs. <11.5 umol/l	115 (6.83):116 (6.95)	1.02 (0.78,1.34)	0.8602
Among patients with SUA ≥ 283 umol/l			
Model 1*: TBIL < 11.5 umol/l vs. ≥11.5 umol/l	174 (28.02):156 (5.96)	6.44 (5.08,8.18)	<0.0001
Model 2†: TBIL < 11.5 umol/l vs. ≥11.5 umol/l	174 (28.02):156 (5.96)	6.45 (5.06,8.23)	<0.0001
Model 3*: TBIL≥11.5 umol/l vs. <11.5 umol/l	156 (5.96):174 (28.02)	0.15 (0.12,0.19)	<0.0001
Model 4†: TBIL ≥ 11.5 umol/l vs. <11.5 umol/l	156 (5.96):174 (28.02)	0.15 (0.12,0.19)	<0.0001

SUA, serum uric acid; TBIL, total bilirubin; CVD, cardiovascular disease; N (%), number of cases (% of number at risk); OR, odds ratios; CI, confidence interval.

^*^Univariable model, not adjusted for any other variables.

^†^Multivariable model, age, duration of diabetes, gender, body mass index, systolic blood pressure, diastolic blood pressure, glycated hemoglobin, low-density lipoprotein cholesterol, high-density lipoprotein cholesterol, triglyceride, self-monitoring, log-transformed urinary albumin to creatinine ratio, and drug use and complications as listed in Table 1,were adjusted in multivariable analysis (Valid sample size = 6707, with 6 missing ACR).

^‡^N (%), number of cases (% of number at risk).

**Table 3:** Interactive effect of SUA and TBIL on CVD in type 2 diabetes.

Exposures	N (%) of CVD‡	OR (95% CI)	P value
Model 1*			
SUA < 283 umol/l and TBIL ≥ 11.5 umol/l	115 (6.83)	1	
SUA ≥ 283 umol/l and TBIL ≥ 11.5 umol/l	156 (5.69)	0.82 (0.64,1.05)	0.1254
SUA < 283 umol/l and TBIL < 11.5 umol/l	116 (6.95)	1.01 (0.78,1.33)	0.8934
SUA ≥ 283 umol/l and TBIL < 11.5 umol/l	174 (28.02)	5.30 (4.10,6.86)	<0.0001
Model 2†			
SUA < 283 umol/l and TBIL ≥ 11.5 umol/l	115 (6.83)	1	
SUA ≥ 283 umol/l and TBIL ≥ 11.5 umol/l	156 (5.69)	0.81 (0.63,1.04)	0.1109
SUA < 283 umol/l and TBIL < 11.5 umol/l	116 (6.95)	0.96 (0.74,1.26)	0.8182
SUA ≥ 283 umol/l and TBIL < 11.5 umol/l l	174 (28.02)	5.18 (4.00,6.72)	<0.0001

SUA, serum uric acid; TBIL, total bilirubin; CVD, cardiovascular disease; N (%), number of cases (% of number at risk); OR, odds ratios; CI, confidence interval.

^*^Univariable model, not adjusted for any other variables.

^†^Multivariable model, age, duration of diabetes, gender, body mass index, systolic blood pressure, diastolic blood pressure, glycated hemoglobin, low-density lipoprotein cholesterol, high-density lipoprotein cholesterol, triglyceride, self-monitoring, log-transformed urinary albumin to creatinine ratio, and drug use and complications as listed in Table 1,were adjusted in multivariable analysis (Valid sample size = 6707, with 6 missing ACR).

^‡^N (%), number of cases (% of number at risk).

**Table 4:** Measures of additive interaction between SUA and TBIL for the risk of CVD.

Measures of interaction	Estimated value	95%CI	P value
Univariable model			
RERI	4.46	3.30–5.62	<0.001
AP	0.84	0.77–0.90	<0.001
S	−27.19	NA	NA
Multivariable model			
RERI	4.40	3.25–5.54	<0.001
AP	0.84	0.77–0.90	<0.001
S	−19.46	NA	NA

SUA, serum uric acid; TBIL, total bilirubin; CVD, cardiovascular disease ; CI, confidence interval; RERI, relative excess risk of interaction; AP, attributable proportion; S, synergy index; NA, not available.

Univariable model: not adjusted for any other variables.

Multivariable model: age, duration of diabetes, gender, body mass index, systolic blood pressure, diastolic blood pressure, glycated hemoglobin, low-density lipoprotein cholesterol, high-density lipoprotein cholesterol, triglyceride, self-monitoring, log-transformed urinary albumin to creatinine ratio, and drug use and complications as listed in Table 1 were adjusted in multivariable analysis (Valid sample size = 6707, with 6 missing ACR).
