# Supplementary material for: Interactive effect of serum uric acid and total bilirubin for cardiovascular disease in Chinese patients with type 2 diabetes
Source: Sci Rep. 2016 Nov 2;6:36437. doi: 10.1038/srep36437 (PMC5090353; doi:10.1038/srep36437)
Supplement: Supplementary Information [file srep36437-s1.doc]

**Interactive effect of serum uric acid and total bilirubin for cardiovascular disease** **in Chinese patients with type 2 diabetes**

Yanfeng Ren(MD), Nan Jin(MD), Tianpei Hong (MD), Yiming Mu (MD), Lixin Guo (MD), Qiuhe Ji(MD), Qiang Li (MD), Xilin Yang (PhD), Linong Ji (MD)

**Appendices**

Supplementary Table 1. Odds ratios of the quartiles levels of SUA and TBIL for CVD in type 2 diabetes

| Exposures | Percentiles percentpercentil | N (%) of CVD^‡^ | OR (95% CI) | P value |
| --- | --- | --- | --- | --- |
| **Models 1*** |  |  |  |  |
| SUA perpercentii | <25^th^ | 83(5.13) | 1 |  |
|  | ≥25^th^-<50^th^ | 148(8.54) | 1.72(1.30, 2.28) | <0.0001 |
|  | ≥50^th^-<75^th^ | 183(11.64) | 2.43(1.86, 3.19) | <0.0001 |
|  | ≥75^th^ | 147(8.22) | 1.65(1.25, 2.18) | <0.0001 |
| TBIL | <25^th^ | 261(16.30) | 1 |  |
|  | ≥25^th^-<50^th^ | 75(4.66) | 0.25(0.19, 0.32) | <0.0001 |
|  | ≥50^th^-<75^th^ | 85(4.69) | 0.25(0.20, 0.32) | <0.0001 |
|  | ≥75^th^ | 140(8.27) | 0.46(0.37, 0.57) | <0.0001 |
| **Model 2†** |  |  |  |  |
| SUA | <25^th^ | 83(5.13) | 1 |  |
|  | ≥25^th^-<50^th^ | 148(8.54) | 1.96(1.47, 2.61) | <0.0001 |
|  | ≥50^th^-<75^th^ | 183(11.64) | 3.08(2.31, 4.12) | <0.0001 |
|  | ≥75^th^ | 147(8.22) | 2.60(1.89, 3.57) | <0.0001 |
| TBIL | <25^th^ | 261(16.30) | 1 |  |
|  | ≥25^th^-<50^th^ | 75(4.66) | 0.25(0.19, 0.32) | <0.0001 |
|  | ≥50^th^-<75^th^ | 85(4.69) | 0.26(0.20, 0.33) | <0.0001 |
|  | ≥75^th^ | 140(8.27) | 0.47(0.37, 0.58) | <0.0001 |

SUA, serum uric acid; TBIL, total bilirubin; CVD, cardiovascular disease; N (%), number of cases (% of number at risk); OR, odds ratios; CI, confidence interval.

^*^ Univariable model, not adjusted for any other variables.

^†^ Multivariable model, age, duration of diabetes, gender, body mass index, systolic blood pressure, diastolic blood pressure, glycated hemoglobin, low-density lipoprotein cholesterol, high-density lipoprotein cholesterol, triglyceride, self-monitoring, log-transformed urinary albumin to creatinine ratio, and drug use and complications as listed in Table 1,were adjusted in multivariable analysis (Valid sample size=6707, with 6 missing ACR).

‡ N (%), number of cases (% of number at risk).

Supplementary Table 2. The perils, the peril ratios and PRISM

| Exposures | CVD | | Peril  (95%CI) | Peril Ratio (95%CI) |
| --- | --- | --- | --- | --- |
|  | Yes | No |  |  |
| SUA < 283 umol/l and TBIL≥11.5 umol/l | 115 | 1568 | 1.07(1.05, 1.08) | 1 |
| SUA ≥ 283 umol/l and TBIL≥11.5 umol/l | 156 | 2584 | 1.06(1.05, 1.07) | 0.98 (0.97, 1.00) |
| SUA < 283 umol/l and TBIL<11.5 umol/l | 116 | 1553 | 1.07(1.06, 1.08) | 1.00 (0.98, 1.01) |
| SUA ≥ 283 umol/l and TBIL<11.5 umol/l l | 174 | 447 | 1.38(1.32, 1.45) | 1.29 (1.24, 1.34) |
| PRISM (95%CI) = 1.30 (1.25, 1.36) |  |  |  |  |

PRISM, peril ratio index of synergy based on multiplicativity; PRISM > 1 indicates a positive interaction between two variables.

Supplementary Table 3. Odds ratios of SUA and TBIL for CVD in type 2 diabetes patients with diabetes duration more than 2 years

| Exposures | N (%) of CVD^‡^ | OR (95% CI) ^†^ | P value |
| --- | --- | --- | --- |
| **All patients** |  |  |  |
| SUA ≥283 umol/l vs. <283 umol/l | 174(8.14):97(6.13) | 1.37(1.06, 1.77) | 0.0162 |
| TBIL<11.5 umol/l vs. ≥ 11.5umol/l | 109(3.93):162(17.16) | 5.06(3.91,6.55) | <0.0001 |
| TBIL≥11.5 umol/l vs.< 11.5umol/l | 109(3.93):162(17.16) | 0.19(0.15, 0.25) | <0.0001 |
| **Among patients with TBIL≥11.5 umol/l** |  |  |  |
| SUA ≥283 umol/l vs. <283 umol/l | 61(3.58):48(4.48) | 0.78(0.53,1.15) | 0.2230 |
| **Among patients with TBIL<11.5 umol/l** |  |  |  |
| SUA ≥283 umol/l vs. <283 umol/l | 113(26.04):49(9.61) | 3.29(2.28, 4.75) | <0.0001 |
| **Among patients with SUA < 283 umol/l** |  |  |  |
| TBIL<11.5 umol/l vs. ≥ 11.5umol/l | 48(4.48):49(9.61) | 2.32(1.53, 3.52) | <0.0001 |
| TBIL≥11.5 umol/l vs.< 11.5umol/l | 48(4.48):49(9.61) | 0.43(0.28, 0.65) | <0.0001 |
| **Among patients with SUA** ≥ **283 umol/l** |  |  |  |
| TBIL<11.5 umol/l vs. ≥ 11.5umol/l | 61(3.58):113(26.04) | 9.55(6.81, 13.40) | <0.0001 |
| TBIL≥11.5 umol/l vs.< 11.5umol/l | 61(3.58):113(26.04) | 0.10(0.07, 0.14) | <0.0001 |

SUA, serum uric acid; TBIL, total bilirubin; CVD, cardiovascular disease; N (%), number of cases (% of number at risk); OR, odds ratios; CI, confidence interval.

^†^ Multivariable model, age, duration of diabetes, gender, body mass index, systolic blood pressure, diastolic blood pressure, glycated hemoglobin, low-density lipoprotein cholesterol, high-density lipoprotein cholesterol, triglyceride, self-monitoring, log-transformed urinary albumin to creatinine ratio, and drug use and complications as listed in Table 1,were adjusted in multivariable analysis (Valid sample size=6707, with 6 missing ACR).

‡ N (%), number of cases (% of number at risk)

Supplementary Table 4. Measures of additive interaction between SUA and TBIL for the risk of CVD in type 2 diabetes patients with diabetes duration more than 2 years

| Measures of interaction | Estimated value | 95%CI | P value |
| --- | --- | --- | --- |
| **Univariable model** |  |  |  |
| RERI | 5.45 | 3.27-7.62 | <0.0001 |
| AP | 0.72 | 0.60-0.84 | <0.0001 |
| S | 6.14 | 2.58-14.56 | <0.0001 |
| **Multivariable model** |  |  |  |
| RERI | 5.45 | 3.25-7.65 | <0.0001 |
| AP | 0.72 | 0.60-0.84 | <0.0001 |
| S | 5.98 | 2.56-13.96 | <0.0001 |

SUA, serum uric acid; TBIL, total bilirubin; CVD, cardiovascular disease ; CI, confidence interval; RERI, relative excess risk of interaction; AP, attributable proportion; S, synergy index.

Univariable model: not adjusted for any other variables.

Multivariable model: age, duration of diabetes, gender, body mass index, systolic blood pressure, diastolic blood pressure, glycated hemoglobin, low-density lipoprotein cholesterol, high-density lipoprotein cholesterol, triglyceride, self-monitoring, log-transformed urinary albumin to creatinine ratio, and drug use and complications as listed in Table 1 were adjusted in multivariable analysis (Valid sample size=6707, with 6 missing ACR).

Supplementary Figure 1. Distribution of CVD cases by exposures to high SUA and low TBIL

Legends: SUA, serum uric acid; TBIL, total bilirubin; CVD, cardiovascular disease.

Supplementary Table 5. Independent effects of SUA and TBIL on CHD, Stroke, PAD and CVD

| Exposures | CHD | | Stroke | | PAD | | CVD | |
| --- | --- | --- | --- | --- | --- | --- | --- | --- |
|  | OR (95% CI) ^†^ |  | OR (95% CI) ^†^ |  | OR (95% CI) ^†^ |  | OR (95% CI) ^†^ |  |
| **All patients** |  |  |  |  |  |  |  |  |
| SUA ≥283 umol/l vs. <283 umol/l | 1.86(1.49,2.32) |  | 1.03(0.70,1.52) |  | 1.18(0.90,1.55) |  | 1.49(1.25,1.78) |  |
| TBIL<11.5 umol/l vs. ≥ 11.5umol/l | 3.16(2.53,3.94) |  | 1.43(0.96,2.11) |  | 1.35(1.02,1.78) |  | 2.15(1.80,2.56) |  |
| TBIL≥11.5 umol/l vs.< 11.5umol/l | 0.31(0.25,0.39) |  | 0.69(0.47,1.03) |  | 0.74(0.56,0.97) |  | 0.46(0.39,0.55) |  |
| **Among patients with TBIL≥11.5 umol/l** |  |  |  |  |  |  |  |  |
| SUA ≥283 umol/l vs. <283 umol/l | 1.12(0.78,1.61) |  | 0.63(0.37,1.07) |  | 0.63(0.44,0.90) |  | 0.80(0.62,1.04) |  |
| **Among patients with TBIL<11.5 umol/l** |  |  |  |  |  |  |  |  |
| SUA ≥283 umol/l vs. <283 umol/l | 5.77(4.30,7.75) |  | 2.57(1.44,4.58) |  | 3.81(2.48,5.87) |  | 5.25(4.06,6.81) |  |
| **Among patients with SUA < 283 umol/l** |  |  |  |  |  |  |  |  |
| TBIL<11.5 umol/l vs. ≥ 11.5umol/l | 1.60(1.11,2.31) |  | 0.79(0.45,1.37) |  | 0.59(0.39,0.90) |  | 0.97(0.74,1.27) |  |
| TBIL≥11.5 umol/l vs.< 11.5umol/l | 0.62(0.43,0.90) |  | 1.26(0.72,2.19) |  | 1.68(1.11,2.55) |  | 1.02(0.78,1.34) |  |
| **Among patients with SUA** ≥ **283 umol/l** |  |  |  |  |  |  |  |  |
| TBIL<11.5 umol/l vs. ≥ 11.5umol/l | 8.43(6.33,11.22) |  | 3.24(1.86,5.65) |  | 3.67(2.51,5.37) |  | 6.45(5.06,8.23) |  |
| TBIL≥11.5 umol/l vs.< 11.5umol/l | 0.11(0.08,0.15) |  | 0.30(0.17,0.53) |  | 0.27(0.18,0.39) |  | 0.15(0.12,0.19) |  |

Abbreviations: SUA, serum uric acid; TBIL, total bilirubin; CVD, cardiovascular disease; CHD, coronary heart disease; PAD, peripheral arterial disease; OR, odds ratios; CI, confidence interval.

† Multivariable model, age, duration of diabetes, gender, body mass index, systolic blood pressure, diastolic blood pressure, glycated hemoglobin, low-density lipoprotein cholesterol, high-density lipoprotein cholesterol, triglyceride, self-monitoring, log-transformed urinary albumin to creatinine ratio, and drug use and complications as listed in Table 1 were adjusted in multivariable analysis.

Supplementary Table 6 Measures of additive interactions between SUA and TBIL for the risk of CHD, Stroke, PAD and CVD

| Measures | CHD | | Stroke | | PAD | | CVD | |
| --- | --- | --- | --- | --- | --- | --- | --- | --- |
|  | Estimate (95%CI) |  | Estimate (95%CI) |  | Estimate (95%CI) |  | Estimate (95%CI) |  |
| Model 1***** |  |  |  |  |  |  |  |  |
| RERI | 7.81(5.12,10.51) |  | 1.72(0.64,2.79) |  | 2.10(1.32,2.88) |  | 4.46(3.30, 5.62) |  |
| AP | 0.81(0.74,0.87) |  | 0.75(0.48,1.02) |  | 0.88(0.72,1.04) |  | 0.84(0.77,0.90) |  |
| S | 10.79(4.37,26.63) |  | -2.85(NA) |  | -1.90(NA) |  | -27.19(NA) |  |
| Model 2**†** |  |  |  |  |  |  |  |  |
| RERI | 7.68(5.03,10.34) |  | 1.62(0.65,2.59) |  | 2.07(1.30,2.84) |  | 4.40(3.25,5.54) |  |
| AP | 0.81(0.75,0.88) |  | 0.79(0.51,1.07) |  | 0.89(0.73,1.04) |  | 0.84(0.77,0.90 |  |
| S | 11.78(4.33,32.03) |  | -1.81(NA) |  | -1.78(NA) |  | -19.46(NA) |  |

Abbreviations: SUA, serum uric acid; TBIL, total bilirubin; CVD, cardiovascular disease; CHD, coronary heart disease; PAD, peripheral arterial
disease; CI, confidence interval.

***** Univariable model, not adjusted for any other variables.

**†** Multivariable model, age, duration of diabetes, gender, body mass index, systolic blood pressure, diastolic blood pressure, glycated hemoglobin, low-density lipoprotein cholesterol, high-density lipoprotein cholesterol, triglyceride, self-monitoring, log-transformed urinary albumin to creatinine ratio, and drug use and complications as listed in Table 1 were adjusted in multivariable analysis.
